# Phase II trial to investigate the safety and efficacy of orally applied niclosamide in patients with metachronous or sychronous metastases of a colorectal cancer progressing after therapy: the NIKOLO trial

**DOI:** 10.1186/s12885-018-4197-9

**Published:** 2018-03-15

**Authors:** Susen Burock, Severin Daum, Ulrich Keilholz, Konrad Neumann, Wolfgang Walther, Ulrike Stein

**Affiliations:** 1 Charité Comprehensive Cancer Center, Invalidenstraße 80, 10117 Berlin, Germany; 2 Department of Medicine I, Gastroenterology, Rheumatology and Infectious Diseases, Charité Universitätsmedizin Berlin, Hindenburgdamm 30, 12200 Berlin, Germany; 3 Experimental and Clinical Research Center, Charité Universitätsmedizin Berlin and Max-Delbrück-Center for Molecular Medicine, Robert-Rössle-Straße 10, 13125 Berlin, Germany; 4 Department for Biostatistics and Clinical Epidemiology, Charité Universitätsmedizin Berlin, Hindenburgdamm 30, 12203 Berlin, Germany; 5 German Cancer Research Center, Im Neuenheimer Feld 280, 69120 Heidelberg, Germany

**Keywords:** Colorectal cancer, Niclosamide, S100A4

## Abstract

**Background:**

Colorectal cancer (CRC) is the second most common cause of all cancer deaths in Europe and the Western world with a lifetime risk of approximately 5%. Despite several improvements in the treatment of patients with unresectable CRC prognosis is poor and there is the need of developing new treatment strategies for patients with metastatic chemorefractory disease.

The S100 calcium binding protein A4 (S100A4) predicts metastasis formation and reduced CRC patient survival. S100A4 was previously identified as transcriptional target of the Wnt/β-catenin signaling pathway. The Food and Drug Administration (FDA)-approved anti-helminthic drug niclosamide is known to intervene in the Wnt/β-catenin pathway signaling, leading to reduced expression of S100A4 linked to restricted in vivo metastasis formation. Thus, we aim at translation of our findings on restricting S100A4-driven metastasis into clinical practice for treating metastasized CRC patients progressing after standard therapy.

**Methods/Design:**

NIKOLO is a phase II, single center, one-arm open-label clinical trial to investigate the safety and efficacy of niclosamide tablets in patients with metastasized CRC progressing under standard therapy. Eligible patients will receive 2 g of orally applied niclosamide once a day and will continue with the treatment once daily till disease progression or toxicity. Toxicities will be graded according to National Cancer Institute Common Terminology Criteria for Adverse Events (NCI CTCAE) v4.03. The primary objective of this trial is to assess the progression free survival after 4 months, secondary objectives are overall survival, time to progression, disease control rate (remission + partial remission + stable disease), and safety. Furthermore, pharmacokinetic analysis will be conducted to evaluate niclosamide plasma concentration.

**Discussion:**

This study is expected to provide evidence of the feasibility, toxicity and efficacy of niclosamide in the treatment of patients with metastasized CRC and could help to establish a new treatment option.

**Trial registration:**

The study is registered with ClinicalTrials.gov (NCT02519582) and the European Clinical Trials Database (EudraCT 2014-005151-20).

## Background

Colorectal cancer (CRC) is the second most common cause of all cancer deaths in Europe and the Western world with a lifetime risk of approximately 5% [1]. Despite the implementation of early detection programs about 20–25% of all patients have already synchronous metastases at the time of diagnosis and approximately 25–50% will develop metastases in the course of disease mainly in liver and lung [2, 3]. Liver metastases are the main cause of death and the only currently available curative therapy is the surgical R0 resection of metastases. Without any further treatment the median survival after metastases detection is poor with about 7.5 month [4].

For patients with unresectable disease several systemic treatment options are available. In recent years prognosis has been improved with median survival rates in clinical trials of up to 30 months [5]. The optimal treatment strategy is highly depending on the aim of the treatment, e.g. prolongation of survival, downsizing with secondary (potentially curative) resection, improving tumor-related symptoms, stopping tumor progression and/or maintaining quality of life, the side effect profile of the regimen, tumor -specific factors and comorbidities of the patient.

Combination chemotherapy regimens with 5-fluorouracil/leucovorin/oxaliplatin (FOLFOX) or 5-fluorouracil/leucovorin/irinotecan (FOLFIRI) provide higher response rates, longer progression-free survival (PFS) and better overall survival (OS) in first-line treatment compared to monotherapy regimens with 5-fluorouracil/leucovorin alone.

For patients with a good performance status and adequate organ function second-line chemotherapy should be offered. The chemotherapy regime in this situation is highly depending on the previous therapies, the therapy-free interval and pre-existing toxicities.

For patients who received an irinotecan-based regimen as first-line treatment the second-line treatment should consist of an oxaliplatin-containing combination (FOLFOX or capecitabine/oxaliplatin). For patients who received an oxaliplatin-based regimen as first-line treatment, the second-line treatment should be irinotecan-based (FOLFIRI or irinotecan alone) [6, 7].

The combination of conventional chemotherapy with molecular targeted therapies such as the anti-vascular endothelial growth factor (anti-VEGF) agents bevacizumab, ramucirumab and aflibercept or the anti-epidermal growth factor receptor (anti-EGFR) agents cetuximab and panitumumab should be considered depending on the tumor biology. After second-line chemotherapy many patients are still in a good condition to receive third-line treatment depending on the organ function and pre-existing toxicities.

Despite the recent improvements in CRC therapy there is still the need of developing new treatment strategies for patients with metastatic chemorefractory disease. One important pathway which is constitutively active in 90% of CRC is the canonical Wnt/β-catenin pathway [8].

The S100 calcium binding protein A4 (S100A4) was identified as a Wnt/β-catenin target gene [9]. S100A4, originally named metastasin 1 (MTS1), is associated with many physiological processes (e.g. migration, invasion, wound healing, neovascularisation). S100A4 is able to induce metastases in different experimental in vitro and animal models [10–13]. S100A4 is overexpressed in many different types of cancer [14, 15]. Its overexpression correlates with poor patients’ prognosis; high S100A4 levels are prognostic for metachronous metastases formation and correlate with reduced patient survival in several cancer types [16–22]. However, S100A4 itself is not tumorigenic as shown by Ambartsumian et al. because transgenic mice with an overexpression of S100A4 do not develop tumors per se [23].

In our own group we identified niclosamide as a transcriptional inhibitor of S100A4 expression by performing a high throughput screen for the human S100A4 promoter–driven reporter gene expression. Niclosamide is used since mid of the 1960s as an anti-helminthic drug which inhibits glucose uptake, oxidative phosphorylation and anaerobic metabolism [24]. Niclosamide is approved by the Food and Drug Administration (FDA) and the European medicines agency (EMA) for treatment of tapeworm infections.

Consequently, we demonstrated the efficacy of niclosamide in cell culture by inhibition of S100A4-induced cell migration and invasion. We identified niclosamide as anti-metastatic drug in colon cancer xenografted mice by restriction of liver metastases linked to shorter survival. In this experimental setting, niclosamide interfered in the Wnt signaling pathway by disrupting the complexation of β-catenin/TCF with the S100A4 promoter leading to reduced expression of S100A4 [13].

Other studies reported that niclosamide also interfered with the Wnt signaling pathways by inhibiting Wnt/Frizzled 1 signaling or by inducing the degradation of the Wnt co-receptor low density lipoprotein receptor-related protein 6 (LRP6) [25, 26]. Osada et al. demonstrated a niclosamide-induced decrease in Dvl2 expression, leading to reduced downstream β-catenin signaling [27]. In hepatocellular cancer cells, niclosamide reduced cell proliferation, induced apoptosis, decreased TOP activity, and lowered β-catenin, Dvl2 and cyclin D1 levels [28]. Ono et al. reported niclosamide-induced reduction of proliferation of primary human leiomyoma cells by inhibiting Wnt/β-catenin pathway activation, such as inhibited TOP activity, down-regulated pathway target genes e.g. Axin2, and reduced nuclear β-catenin translocation [29]. Londoño-Joshi et al. demonstrated niclosamide-reduced levels of LRP6 and β-catenin for basal-like breast cancers [30]. For ovarian cancer, niclosamide treatment resulted in increased cytotoxicity, reduction of Wnt/β-catenin signaling by TOP assay, and decreased Wnt pathway proteins e.g. Axin2 and cyclin D1 [31]. King et al. demonstrated niclosamide-induced abrogation of Wnt7A/β-catenin signaling, inhibition of β-catenin transcriptional activity, cell viability, and decrease in cell migration [32]. Satoh et al. showed that niclosamide inhibited cell proliferation, induced caspase-dependent apoptosis and G1 cell cycle arrest, and decreased cell migration, reduced the level of mediators of epithelial-to-mesenchymal transition, and decreased β-catenin expression, [33]. Functionally, niclosamide is able to block further pathways besides Wnt/β-catenin signaling, known to be decisive for cancer initiation, progression and metastases [34, 35]. For instance, it inhibits the mTORC1 activity by lysosomal dysfunction in different cancer types [36–38], blocks the STAT3 phosphorylation and its translocation to the nucleus in prostate cancer cells [39], and inhibits the NF-κB and Notch pathways [40, 41]. Thus, niclosamide has been reported as an anti-cancer molecule in numerous cancer types like CRC [13], osteosarcoma [42], breast cancer [30, 37], ovarian cancer [31, 43], prostate cancer [44], non-small cell lung cancer (NSCLC) [45, 46], glioblastoma [40], head and neck cancer [47], multiple myeloma, and leukemia [41, 48].

Niclosamide has only little side effects and seems to be well tolerated even when applied over a long period [49]. The oral dose of niclosamide for adults in anti-helminthic treatments is 2 g on day one followed by 1 g daily for 6 consecutive days. The serum concentration of niclosamide after a single dose of 2 g leads to maximal serum concentrations of 0.25–6.0 μg/ml which corresponds to the concentration used on the above mentioned models [34, 49].

As there is strong evidence from in vitro and in vivo experiments that niclosamide might be beneficial as anti-tumorigenic and anti-metastatic drug we designed the hereafter described clinical trial to investigate the safety and efficacy of orally applied niclosamide in patients with metachronous or sychronous metastases of a CRC who progressed after therapy and for whom no other standard therapy is available.

## Methods/Design

### Study aims

This study evaluates the safety and efficacy of orally applied niclosamide in patients with metachronous or synchronous metastases of CRC origin, who progressed under previous therapy.

### Study objectives

Primary objective is PFS after 4 months. PFS after 4 months will be evaluated according to the Response Evaluation Criteria In Solid Tumors (RECIST) Version 1.1.

Secondary objectives are OS, time to progression, disease control rate (remission + partial remission + stable disease), and safety.

Pharmacokinetic analysis will be done to investigate niclosamide plasma concentration and a potential correlation with efficacy.

### Study design

The study is a phase II single center, one-arm open-label clinical trial for patients with metastatic CRC.

### Study schedule

The trial has started on August 2015. The estimated accrual duration is 30 months. The estimated study completion date is 2018 (final data collection, date for primary outcome measure). Survival status will be collected for 2 years after registration.

### Trial organization

The trial is organised by the Charité Comprehensive Cancer Center (CCCC) in collaboration with the Max-Delbrück-Center for Molecular Medicine (MDC), Berlin, Germany.

### Statistics

The study is a one-arm open-label clinical trial with interim analyses for futility of patients with metastatic CRC.

A Simon two-stage design will be used to allow early termination due to futility. The null hypothesis is that the probability of being free of progression (having stable disease, partial or complete response) at 4 months is p_0_ = 0.25 (H_0_: p < =p_0_ = 0.25) estimated according to the recent literature [50, 51]. This null hypothesis will be tested with the Binomial test one-sided against the alternative H_1_ (estimated progression-free probability at 4 months of the experimental therapy) p1 = 0.5. Samples size for both stages were calculated according to the optimal design after Simon [52].

In the first stage 17 patients will be recruited. If less than 5 of the first 17 patients are progression-free at 4 months the trial will be terminated prematurely. Otherwise 20 additional patients will be enrolled (up to a total of 37 patients).

The null hypothesis will be rejected if at least 13 patients out of 37 will be free of progression at 4 months. This design (optimal design after Simon) yields a type I error rate of 0.046 and a power of 0.902.

Analyses of PFS and OS survival will performed with Kaplan-Meier-curves, hazard ration will be reported with 95% confidence intervals. For safety analysis the worst toxicities in each cycle will be reported according to the National Cancer Institute (NCI) Common Terminology Criteria for Adverse Events (CTCAE) Version 4.03. A descriptive analysis of adverse events (AEs) and serious adverse events (SAEs) will be provided.

### Recruitment process

The trial is expected to take up to 30 months to complete recruitment. Participants will be recruited from the Charité Universitätsmedizin Berlin. Following verbal and written study information in form of the patient information sheet and extensive discussion with a clinical trial physician, patients will get at least 24 h time to consider participation.

Patients who meet all the inclusion criteria and none of the exclusion criteria listed in Table 1 will be invited to provide written informed consent.

**Table 1:** Nikolo trial inclusion and exclusion criteria

**Inclusion criteria**
Disease related
Patients with metachronous or synchronous metastases of a colorectal cancer progressing under standard therapy
No proven brain metastases
No curative option available
No standard therapy available
At least one metastasis has to be measurable according to RECIST V 1.1 in X-ray computed tomography or magnetic resonance imaging (not older than 2 weeks before inclusion into the trial)
Demographic
Age > 18 years
Eastern Cooperative Oncology Group performance status ECOG 0–1
Laboratory
Absolute neutrophil count ≥ 1.5 × 10^9^/ L
Platelet count ≥ 100 × 10^9^/L
Leukocytes ≥ 1.0 × 10^9^/L
Hemoglobin ≥ 9.0 g/dL or 5.59 mmol/l
Bilirubin ≤ 2 x Upper Limit of Normal (ULN) if not due to Gilbert’s syndrome
AST ≤ 2.5xULN in patients without liver metastases or ≤ 5.0xULN in patients with liver metastases
ALT ≤ 2.5xULN in patients without liver metastases or < 5.0xULN in patients with liver metastases
Adequate renal function (creatinine ≤ 1.5xULN)
Ethical/Other
Electrocardiography without clinical significant abnormalities
Written informed consent before inclusion according to the ICH-GCP and national/local regulations
Patients should use adequate birth control measures, during the study treatment period and for at least 3 months after the last study treatment
Women of childbearing potential need to have a negative pregnancy test 72 h before the application of the first dose of niclosamide
Patients who are breastfeeding must stop breastfeeding before the first dose of the study drug and not restart till 8 week after the last drug intake
**Exclusion criteria**
Concurrent conditions
Pregnant or lactating females
Other malignant disease (except colorectal cancer) within the last 5 years before inclusion in the trial except adequately treated basal cell carcinoma of the skin or squamous cell carcinoma of the skin, in situ carcinoma of the cervix. Patients with a malignant disease in history have to be free of disease for 5 years
Clinical significant heart disease like e.g. uncontrolled blood pressure; heart failure NYHA grade > 2; cardiac infarction within the last 12 months
Known uncontrolled concomitant disease despite treatment like e.g. chronic obstructive pulmonary disease
Known alcohol or drug abuse
Serious infection
Any psychological, familial, sociological or geographical condition, potentially hampering compliance with the study protocol and follow-up schedule
Ethical/Other
Participation in another interventional study within the last 30 days
Known hypersensitivity against a part of the study drug
Life expectancy < 3 months
Human immunodeficiency virus infection or active hepatitis B/C
Patients that are committed to an institution by official or judicial order
Persons that are depending on the sponsor, investigator or deputy

### Study intervention and general considerations for dose modifications

Patients will start the treatment with the oral intake of 2 g niclosamide on day 1 and will continue with the medication once daily at the same time after a meal till disease progression or toxicity. Toxicities will be graded according to NCI CTCAE v4.03.

In case of an allergic reaction or vomiting ≥ grade II or diarrhoea or nausea or any other toxicity ≥ grade 3 the study medication will be paused until recovery and consequently reduced by one dose level (500 mg). If dose reductions to levels less than 1 g or discontinuation of the study treatment longer than 3 weeks are necessary, the study treatment will be terminated.

### Assessments

Patients will be followed up closely for side effects. This includes physical examinations as well as blood tests. Additional intensive analyses of niclosamide levels in the blood will be conducted in the first patient cohort (patients 1–5) in the first week to examine the absorption of niclosamide from the intestine and systemic circulation. To assess efficacy imaging will be performed every 2 months to evaluate the disease status.

### Translational research

Patients will be asked to consent for optional translational research including analysis of patients’ tumor tissue and additional blood analysis as described hereunder.

#### Analysis of S100A4 expression in patient primary tumors and/or metastases or recurrent tumors

If available, tumor tissue of the primary tumor and/or metastasis or recurrent tumor will be collected for patients who consent for optional translational research to do further analyses in specific subgroups (responder/non-responder). These tissues will be analyzed for S100A4 expression. These initial analyses characterizing the molecular starting point of the patient, depend on the availability of the amount of tissue procured for research purposes.

#### Analysis of potential niclosamide-induced effects on circulating S100A4 transcripts in patient plasma

The following initial analyses for characterization of the molecular starting point of the patient will be performed by using patients’ plasma for determination of the basal levels of circulating S100A4 transcripts.

Peripheral blood samples will be collected at baseline and at end of treatment (due to progression, toxicity or other reasons) for all patients. For patients who consented for the optional translational research additional blood samples will be collected during routine blood taking at week 1, week 4 and thereafter monthly until progression. Blood samples will be used to discover and validate the prognostic and predictive value of S100A4 as well as for response to niclosamide and to evaluate relationship between other clinical outcome parameters.

#### Plasma generation

Immediately after every blood taking, plasma will be generated. Blood initially collected in EDTA tubes will be processed in the lab for generation of plasma aliquots (400 μl volume) which will be stored frozen at − 80 °C. Analysis will be performed after completion of collection of all samples.

Additional analyses of further circulating transcripts such as beta-catenin might follow in patients who consented for the optional translational research.

#### Determination of niclosamide plasma concentration

Plasma samples (400 μl) are supplemented with acetonitrile (800 μl) containing naproxen (0.25 μg/ml). The mixture is vortexed for 30 s and centrifuged at 15000 g for 20 min at room temperature. An aliqoute of 800 μl of supernatant is withdrawn and evaporated to dryness (N2, 50 °C). The samples were reconstituted in 400 μl dilution solvent consisting of acetonitrile/methanol/water (5%/5%/90%, *v*/v/v) and subjected to HPLC.

HPLC (Shimadzu) will be performed with Trentec Select columns (RP C8 120 ODS3 5 μm; 125 × 3,0 mm with guard column; mobile phase A: acetonitrile/water/phosphoric acid (70/30/0.1, *v*/v/v); mobile phase B: acetonitrile/water/phosphoric acid (25/75/0.1, v/v/v); gradient: 0.01 min B 70%; 8 min B 70%; 8.1 min B 0%; 25 min B 0%; 25.1 min B 70%; flow rate: 0.5 ml/min). Time of analysis is 50 min, injector volume is 100 μl, detection l ABS [nm] is performed at 250 nm.

HPLC calibration curves of naproxen and niclosamide are linear in the range of 0.05 to 1 μg/ml concentration of each analyte of a dilution series in dilution solvent (= acetonitrile/methanol/water (5%/5%/90%, v/v/v). The parameters of a linear regression equation are as follows, naproxen: R2 = 0.9999, y = 216,017×; niclosamide: R2 = 0.9937, y = 230,993× confirming a good linearity for both analytes.

Determination of niclosamide in human plasma samples is done by HPLC (BioTez GmbH, Berlin).

## Discussion

Several research groups and our own studies could show that niclosamide inhibits crucial pathways associated with proliferation, migration and invasion like the Wnt/β-catenin pathway, leading to anti-cancer activity of the drug. Niclosamide is initially EMA- and FDA-approved for anti-helminthic treatment and has a good safety profile, which creates a solid basis for repositioning this drug for alternate uses. Our preclinical studies supported the strong potential of niclosamide as anti-cancer drug, which interferes with tumor progression and metastases formation via S100A4 inhibition. However, its application in cancer patients has not been investigated in patients yet. Therefore, the use of niclosamide represents a novel approach of particularly metastases intervention via inhibition of a defined molecular target, such as S100A4.

To better explore the therapeutic potential of niclosamide there is the need to conduct a clinical trial to investigate the safety, efficacy and pharmacokinetics of orally applied niclosamide in cancer patients, and most importantly, to investigate if niclosamide treatment will result in prolongation of the PFS of the patients. The attractiveness of this study is based on the fact that the drug activity in patients will be correlated with the molecular analysis of the target molecule S100A4 in patient’s blood as the easiest accessible liquid biopsy for the timely monitoring. Furthermore, potential limiting factors, such as possible intra-individual difference in the drug absorption rate, will provide important additional information to what extend this will lead to variations of its anti-cancer efficacy.

As there is currently no predictive biomarker for response to niclosamide available we included an intense translational research program in our study to gain deeper insights in the mechanisms of niclosamide mediated anti-cancer activity. Thus, exploring the therapeutic potential of niclosamide in colon cancer patients in correlation to its target molecule S100A4 will enable us to evaluate this approach for broader uses in cancer therapy, where Wnt-signaling interference represents a rationale.

## Abbreviations

CRC: Colorectal cancer
CTCAE: Common Terminology Criteria for Adverse Events
EMA: European medicines agency
FDA: Food and Drug Administration
FOLFIRI: 5-fluorouracil/leucovorin/irinotecan
FOLFOX: 5-fluorouracil/leucovorin/oxaliplatin
ICH-GCP: International Conference on Harmonisation about Good Clinical Practice guidelines
LRP6: Low density lipoprotein receptor-related protein 6
MTS1: Metastasin 1
NCI: National Cancer Institute
OS: Overall survival
PFS: Progression-free survival
RECIST: Response Evaluation Criteria In Solid Tumors
S100A4: S100 calcium binding protein A4
ULN: Upper Limit of Normal

## References

[1] Ferlay J, Steliarova-Foucher E, Lortet-Tieulent J (2013). Cancer incidence and mortality patterns in Europe: estimates for 40 countries in 2012. Eur J Cancer.

[2] Schmoll HJ, Van CE, Stein A (2012). ESMO Consensus Guidelines for management of patients with colon and rectal cancer. A personalized approach to clinical decision making. Ann Oncol.

[3] Kanas GP, Taylor A, Primrose JN (2012). Survival after liver resection in metastatic colorectal cancer: review and meta-analysis of prognostic factors. Clin Epidemiol.

[4] Stangl R, Altendorf-Hofmann A, Charnley RM (1994). Factors influencing the natural history of colorectal liver metastases. Lancet.

[5] Van CE, Cervantes A, Nordlinger B (2014). Metastatic colorectal cancer: ESMO Clinical Practice Guidelines for diagnosis, treatment and follow-up. Ann Oncol.

[6] Rougier P, Van CE, Bajetta E (1998). Randomised trial of irinotecan versus fluorouracil by continuous infusion after fluorouracil failure in patients with metastatic colorectal cancer. Lancet.

[7] Seymour MT, Maughan TS, Ledermann JA (2007). Different strategies of sequential and combination chemotherapy for patients with poor prognosis advanced colorectal cancer (MRC FOCUS): a randomised controlled trial. Lancet.

[8] Giles RH, van Es JH, Clevers H (2003). Caught up in a Wnt storm: Wnt signaling in cancer. Biochim Biophys Acta.

[9] Stein U, Arlt F, Walther W (2006). The metastasis-associated gene S100A4 is a novel target of beta-catenin/T-cell factor signaling in colon cancer. Gastroenterology.

[10] Stein U, Arlt F, Smith J (2011). Intervening in beta-catenin signaling by sulindac inhibits S100A4-dependent colon cancer metastasis. Neoplasia.

[11] Dahlmann M, Okhrimenko A, Marcinkowski P (2014). RAGE mediates S100A4-induced cell motility via MAPK/ERK and hypoxia signaling and is a prognostic biomarker for human colorectal cancer metastasis. Oncotarget.

[12] Sack U, Walther W, Scudiero D (2011). S100A4-induced cell motility and metastasis is restricted by the Wnt/beta-catenin pathway inhibitor calcimycin in colon cancer cells. Mol Biol Cell.

[13] Sack U, Walther W, Scudiero D (2011). Novel effect of antihelminthic Niclosamide on S100A4-mediated metastatic progression in colon cancer. J Natl Cancer Inst.

[14] Schneider M, Hansen JL, Sheikh SP (2008). S100A4: a common mediator of epithelial-mesenchymal transition, fibrosis and regeneration in diseases?. J Mol Med (Berl).

[15] Helfman DM, Kim EJ, Lukanidin E (2005). The metastasis associated protein S100A4: role in tumour progression and metastasis. Br J Cancer.

[16] Boye K, Nesland JM, Sandstad B (2012). EMMPRIN is associated with S100A4 and predicts patient outcome in colorectal cancer. Br J Cancer.

[17] Rud AK, Lund-Iversen M, Berge G (2012). Expression of S100A4, ephrin-A1 and osteopontin in non-small cell lung cancer. BMC Cancer.

[18] Boye K, Nesland JM, Sandstad B (2010). Nuclear S100A4 is a novel prognostic marker in colorectal cancer. Eur J Cancer.

[19] Boye K, Maelandsmo GM (2010). S100A4 and metastasis: a small actor playing many roles. Am J Pathol.

[20] Natarajan J, Hunter K, Mutalik VS (2014). Overexpression of S100A4 as a biomarker of metastasis and recurrence in oral squamous cell carcinoma. J Appl Oral Sci.

[21] Huang H, Zheng HY, Liu ZL (2014). Prognostic significance of relaxin-2 and S100A4 expression in osteosarcoma. Eur Rev Med Pharmacol Sci.

[22] Stein U, Burock S, Herrmann P (2011). Diagnostic and prognostic value of metastasis inducer S100A4 transcripts in plasma of colon, rectal, and gastric cancer patients. J Mol Diagn.

[23] Ambartsumian NS, Grigorian MS, Larsen IF (1996). Metastasis of mammary carcinomas in GRS/A hybrid mice transgenic for the mts1 gene. Oncogene.

[24] Weinbach EC, Garbus J (1969). Mechanism of action of reagents that uncouple oxidative phosphorylation. Nature.

[25] Chen M, Wang J, Lu J (2009). The anti-helminthic niclosamide inhibits Wnt/Frizzled1 signaling. Biochemistry.

[26] Chen W, Chen M, Barak LS (2010). Development of small molecules targeting the Wnt pathway for the treatment of colon cancer: a high-throughput screening approach. Am J Physiol Gastrointest Liver Physiol.

[27] Osada T, Chen M, Yang XY (2011). Antihelminth compound niclosamide downregulates Wnt signaling and elicits antitumor responses in tumors with activating APC mutations. Cancer Res.

[28] Tomizawa M, Shinozaki F, Motoyoshi Y (2013). Niclosamide suppresses hepatoma cell proliferation via the Wnt pathway. Onco Targets Ther.

[29] Ono M, Yin P, Navarro A (2014). Inhibition of canonical WNT signaling attenuates human leiomyoma cell growth. Fertil Steril.

[30] Londono-Joshi AI, Arend RC, Aristizabal L (2014). Effect of niclosamide on basal-like breast cancers. Mol Cancer Ther.

[31] Arend RC, Londono-Joshi AI, Samant RS (2014). Inhibition of Wnt/beta-catenin pathway by niclosamide: a therapeutic target for ovarian cancer. Gynecol Oncol.

[32] King ML, Lindberg ME, Stodden GR (2015). WNT7A/beta-catenin signaling induces FGF1 and influences sensitivity to niclosamide in ovarian cancer. Oncogene.

[33] Satoh K, Zhang L, Zhang Y (2016). Identification of niclosamide as a novel anticancer agent for adrenocortical carcinoma. Clin Cancer Res.

[34] Li Y, Li PK, Roberts MJ (2014). Multi-targeted therapy of cancer by niclosamide: a new application for an old drug. Cancer Lett.

[35] Pan JX, Ding K, Wang CY (2012). Niclosamide, an old antihelminthic agent, demonstrates antitumor activity by blocking multiple signaling pathways of cancer stem cells. Chin J Cancer.

[36] Li M, Khambu B, Zhang H (2013). Suppression of lysosome function induces autophagy via a feedback down-regulation of MTOR complex 1 (MTORC1) activity. J Biol Chem.

[37] Balgi AD, Fonseca BD, Donohue E (2009). Screen for chemical modulators of autophagy reveals novel therapeutic inhibitors of mTORC1 signaling. PLoS One.

[38] Fonseca BD, Diering GH, Bidinosti MA (2012). Structure-activity analysis of niclosamide reveals potential role for cytoplasmic pH in control of mammalian target of rapamycin complex 1 (mTORC1) signaling. J Biol Chem.

[39] Ren X, Duan L, He Q (2010). Identification of niclosamide as a new small-molecule inhibitor of the STAT3 signaling pathway. ACS Med Chem Lett.

[40] Wieland A, Trageser D, Gogolok S (2013). Anticancer effects of niclosamide in human glioblastoma. Clin Cancer Res.

[41] Jin Y, Lu Z, Ding K (2010). Antineoplastic mechanisms of niclosamide in acute myelogenous leukemia stem cells: inactivation of the NF-kappaB pathway and generation of reactive oxygen species. Cancer Res.

[42] Liao Z, Nan G, Yan Z (2015). The anthelmintic drug niclosamide inhibits the proliferative activity of human osteosarcoma cells by targeting multiple signal pathways. Curr Cancer Drug Targets.

[43] Yo YT, Lin YW, Wang YC (2012). Growth inhibition of ovarian tumor-initiating cells by niclosamide. Mol Cancer Ther.

[44] Lu W, Lin C, Roberts MJ (2011). Niclosamide suppresses cancer cell growth by inducing Wnt co-receptor LRP6 degradation and inhibiting the Wnt/beta-catenin pathway. PLoS One.

[45] Li R, Hu Z, Sun SY (2013). Niclosamide overcomes acquired resistance to erlotinib through suppression of STAT3 in non-small cell lung cancer. Mol Cancer Ther.

[46] Stewart RL, Carpenter BL, West DS (2016). S100A4 drives non-small cell lung cancer invasion, associates with poor prognosis, and is effectively targeted by the FDA-approved anti-helminthic agent niclosamide. Oncotarget.

[47] Li R, You S, Hu Z (2013). Inhibition of STAT3 by niclosamide synergizes with erlotinib against head and neck cancer. PLoS One.

[48] Khanim FL, Merrick BA, Giles HV (2011). Redeployment-based drug screening identifies the anti-helminthic niclosamide as anti-myeloma therapy that also reduces free light chain production. Blood Cancer J.

[49] Andrews P, Thyssen J, Lorke D (1982). The biology and toxicology of molluscicides, bayluscide. Pharmacol Ther.

[50] Grothey A, Van CE, Sobrero A (2013). Regorafenib monotherapy for previously treated metastatic colorectal cancer (CORRECT): an international, multicentre, randomised, placebo-controlled, phase 3 trial. Lancet.

[51] Van CE, Tabernero J, Lakomy R (2012). Addition of aflibercept to fluorouracil, leucovorin, and irinotecan improves survival in a phase III randomized trial in patients with metastatic colorectal cancer previously treated with an oxaliplatin-based regimen. J Clin Oncol.

[52] Simon R (1989). Optimal two-stage designs for phase II clinical trials. Control Clin Trials.

